# Perceptions of being grandparents of children living with type 1 diabetes in Sweden: a qualitative interview study

**DOI:** 10.3389/fcdhc.2026.1763046

**Published:** 2026-04-02

**Authors:** Åsa Carlsund, Sara Olsson, Åsa Hörnsten

**Affiliations:** Department of Nursing, Umeå University, Umeå, Sweden

**Keywords:** grandchildren, grandparents, perceptions, qualitative content analysis, type 1 diabetes

## Abstract

**Aim:**

This study aimed to describe the perceptions of being grandparents of a grandchild living with type 1 diabetes.

**Introduction:**

When a child develops a lifelong condition, it affects the whole family, and caring for a child with chronic illness can be stressful. This ongoing stress can impact family relationships. In addition to parents, siblings and grandparents are often among the first to learn about the child’s condition and may be the initial source of support.

**Methods:**

Individual interviews were conducted with 11 grandparents from northern Sweden who met the inclusion criteria of having a grandchild with type 1 diabetes. The data were analyzed using qualitative content analysis.

**Results:**

The analysis of the interviews uncovered one main theme: being the lighthouse that lights up dark paths but still needing attention to keep shining. Two themes emerged: facing and managing the unknown and balancing expectations and personal needs. Each theme included three subthemes: understanding an emotionally overwhelming situation, taking on new responsibilities and pushing boundaries, and using but not fully trusting self-management technologies. Other aspects included handling a growing pile of concerns, trying to keep the family united, and maintaining personal activities.

**Conclusion:**

Grandparents play an essential role in families with children who have type 1 diabetes. They assume significant responsibilities by learning self-management technologies and providing emotional and practical support, which are vital for family stability and the child’s well-being. However, this role presents challenges, such as fears of making mistakes in diabetes management and worries about their child’s and grandchild’s struggles. Despite these challenges, many grandparents show resilience and are eager to support their families. They remain actively involved, primarily relying on informal support and information from their children, partners, or patient organizations.

## Introduction

The entire family is affected when a child develops a lifelong condition and taking care of a child with lifelong diseases can be extremely stressful. This ongoing stress may affect the family dynamic (1, 2). In addition to parents, siblings, and grandparents may be among the first to be informed about their grandchild’s lifelong condition and are often the first to offer the family support (3, 4).

The lifelong condition, type 1 diabetes can develop at any age, but the onset is prevalent in children and adolescents (5). Treatment includes insulin administration, blood glucose monitoring, and carbohydrate counting, all affected by factors such as growth, exercise, diet, stress, and sleep (6–8). Type 1 diabetes self-management also involves problem solving, risk reduction, and healthy coping strategies (9). In recent years, though, significant technological advancements in self-management have emerged, including innovations such as continuous glucose monitoring, sensors, alarms, and insulin pumps (8, 10) but which may need updated knowledge to manage.

In the general population, grandparents often offer a diverse range of care and support (11). However, when a child faces a lifelong disease such as type 1 diabetes, the dynamics might change, presenting more significant challenges for the entire family (1, 2, 4). Grandparents’ involvement with grandchildren varies significantly across the globe (12). While some grandparents may not live with their grandchildren, they still play significant roles through social interaction, guidance, as well as emotional and financial support (13, 14). They often provide emotional support and practical assistance, and especially during unexpected events such as family crises, parental divorce, or family health issues (15).

At the end of 2021, approximately 2.2 million grandparents comprised Sweden’s population. Grandparents had 3.3 million grandchildren residing in Sweden (Statistics Sweden, 2022). Grandparenthood can be understood from social, familial, and individual perspectives. Changes in society, have influenced the roles and expectations of grandparents. As noted by Pulgaron et al. (16), family dynamics can vary widely, shaped by factors such as cultural norms and family structure (17). On an individual level, grandparenthood can offer meaningful relationships (18). Some evidence indicates that supportive grandparents can positively influence the health outcomes of children (4, 19) and particularly when there are a child with type 1 diabetes in the family. However, the direction of their influence remains unclear and varies across different health areas and cultures (16).

### Aim

The aim of this study was to describe the perceptions of being grandparents of children living with type 1 diabetes in Sweden.

## Material and methods

### Design

This study used a qualitative approach, reporting results from interviews with grandparents of children living with type 1 diabetes. The study was carried out within a constructivist–interpretivist paradigm, which holds that reality is socially constructed and that knowledge is exchanged between participants and researchers. This paradigm shaped the study design, data collection, and analysis methods, focusing on understanding participants’ personal experiences (20). To support the interpretation of the findings, the study was also guided by a systems-oriented perspective, drawing on Bronfenbrenner’s ecological systems theory (21). This viewpoint emphasizes how individuals’ experiences are influenced by interactions within and between family subsystems, which align with the grandparents’ role as secondary and important caregivers.

### Participants

In total, sixteen participants were interested, five of them withdrew due to time constraints or illness. Eleven grandparents from northern Sweden chose to participate in the study, where nine were grandmothers and two grandfathers. **Table 1** presents the participants’ ages ranging from 59 to 72 (mean age of 67 years). Eight had a university education, and all participants were married or cohabiting. Two participants lived in urban areas, while nine lived in rural areas. The 11 participants were all grandparents of a child with type 1 diabetes, offering insights from this specific family role. None of the participants was co-living with their grandchild. Eight had daily contact, eight often had their grandchildren stay overnight, and three rarely or never had their grandchildren stay overnight, nor did they have daily contact.

**Table 1:** Participant characteristics (N = 11).

Characteristic	Category	n	Range / Mean
Age	–	–	59–72 (M = 67)
Gender	Grandmother	9	–
	Grandfather	2	–
Education	High school	3	–
	University	8	–
Place of living	Rural	9	–
	Urban	2	–

M = mean.

### Data collection

This research contributes to a larger study, and the grandparents were recruited from a coordinated meeting with a patient organization. All participants attending the meeting received study information, and those interested in learning more about participation were asked to provide their contact details so that the first author could contact them later. Before participating in this study, all participants were given both oral and written information about its aims and procedures and provided informed consent. Data collection took place from May to June 2025. The individual interviews lasted between 36 and 50 minutes and were recorded via Microsoft Teams. An interview guide containing background questions and open-ended questions was applied. The open-ended questions started with “can or would you please tell me about…” Questions asked were; …” when your grandchild was diagnosed with T1D”… What is working well/unwell right now, when the child is with you, … “family relationships have been affected by T1D, … balance your own life when T1D is present, … what kind of expectations are there of you as a grandparent, …worries and stress, and what type of support/knowledge about T1D would you like to have? We thought we had collected enough data to pinpoint key differences in experiences (22, 23) by the tenth interview; however, we conducted an additional interview to see if more data would improve our understanding. It was concluded that these extra interviews did not increase our knowledge of the topic.

### Analysis

The interview transcripts underwent qualitative content analysis according to Graneheim and Lundman (22) and Lindgren et al. (23). The initial phase involved decontextualization, where the transcribed text was read several times to develop a general understanding before splitting it into meaning units relevant to the study aim. These meaning units were then condensed to preserve their core content and labeled with codes that captured their essential characteristics. In the re-contextualization phase, the codes were compared and organized into groups based on their similarities and differences. The analysis continued to organize preliminary themes into subthemes and themes based on interpretation and abstraction. The analysis was a dynamic process with movements back and forth between transcribed interviews and levels of interpretation and abstraction.

### Trustworthiness

A systematic approach was employed to analyze the interviews (22, 23). This process included de-contextualization and re-contextualization to ensure a thorough review and categorization of the data while preserving the integrity of the parents’ responses. Credibility was demonstrated by providing representative quotations from the transcribed interviews, which showed direct examples of the participants’ perceptions. Interviews always include multiple meanings (22). Regarding dependability, there is consensus among co-researchers that the interpretations reflected the participants’ perspectives. Details regarding the participants’ characteristics, along with the data collection and analysis methodologies, were included to ensure transferability. Presenting results with quotations enables readers to assess the relevance of these results in other contexts.

### Ethical considerations

The ethical standards set forth by the Helsinki Declaration and the World Medical Association (WMA, 2022) were adhered to, receiving approval from the Swedish Ethical Review Authority (XXX). Participation was voluntary, and all participants were aware of their right to opt out. Written informed consent was obtained from the participants. We made efforts to ensure pseudonymity throughout the research process. Each consent form was assigned a unique digit corresponding to each participant, and these identifiers were securely maintained during all phases of the research process.

## Results

### Being the lighthouse that lights up dark paths, but still in need of attention to keep shining

The perceptions of being a grandparent to a child living with type 1 diabetes was described as being the lighthouse that lights up dark paths, but still in need of attention to keep shining. The grandparents faced and managed the unknown, interpreted as lighting up dark paths. They balanced expectations grounded within themselves and from family members with their own needs, illustrated as the lighthouse in need attention to keep shining.

An overview of the results is presented in **Table 2**.

**Table 2:** Results presented as one main theme, two themes, and six subthemes.

Main theme	Themes	Subthemes
Being the lighthouse that lights up dark paths, but still in need of attention to keep shining	Facing and managing the unknown	Grasping an emotionally overwhelming situation
		Taking on new responsibilities and pushing margins
		Using but not fully trusting self-management technologies
	Balancing expectations and own needs	Handling a growing pile of concerns
		Trying to hold the family together
		Maintaining personal activities

Themes and subthemes illustrate grandparents’ experiences of supporting a grandchild living with type 1 diabetes.

### Facing and managing the unknown

The grandparents had to face and manage the unknown. Most grandparents described this early phase as emotionally overwhelming, marked by shock, uncertainty, and a deep sense of vulnerability. Unlike parents, who were involved in ongoing daily care, grandparents experienced the situation from a step removed, which caused emotional strain and limited opportunities to build confidence in diabetes management.

#### Trying to grasp an emotionally overwhelming situation

For the grandparents, it was an emotionally crushing situation. Within hours, their grandchild’s health had taken a devastating turn. One moment, everything was fine, and the next, their lives were turned upside down. At first, they couldn’t process what was happening. It took time for the reality to set in, leaving them in shock and sorrow.

“When we first heard what had happened, it felt unreal. You know it’s serious, but it takes time before your mind catches up.” (Grandmother, 68).

The entire family was profoundly affected, each member dealing with the situation in their own way. The grandparents struggled to understand the seriousness of the illness and the rapid changes it brought to their lives. The emotional toll was substantial, and they had to accept this new reality, supporting one another and their family members.

“It wasn’t like a dark hole, but you didn’t really understand what it was about from the beginning. Even if you had close acquaintances with diabetes, it was just that it got this close and it was so dramatic. He was admitted to the hospital, and it felt like you were just breathing in and out the whole time, which was a bit scary”. (Grandmother, 63 years).

Grandparent expressed sadness about their grandchild being forced to live with a serious lifelong illness, which carries the risk of both short- and long-term complications such as low blood glucose, blindness, and amputation, and in addition to that, throughout life wear a sensor and an insulin pump.

“The small body dragging along an insulin pump, and I am not able to solve the problem like I solve helping her with the puzzle pieces”. (Grandfather, 66 years).

#### Taking on new responsibilities and pushing margins

The grandparents embraced new roles and pushed their boundaries. Several explained that they faced their own fears, especially around insulin dosing and the risk of errors. A few mentioned that, unlike parents, they didn’t have many chances to practice, which made them feel more insecure. However, several grandparents challenged their own limitations and pushed themselves past doubts and fears of making mistakes that could negatively affect their grandchild. For instance, one grandparent shared their fear of incorrectly dosing insulin, worrying that even a small error could lead to serious health consequences for the grandchild. Despite initial fears of making mistakes, a sense of security emerged over time. After some time, the grandparents considered their theoretical knowledge of diabetes management to be reasonably good.

“I wanted to help more, but I was terrified of making a mistake with the insulin. It’s a heavy responsibility when you don’t do it every day.” (Grandmother, 67).

The grandparents acknowledged that although it was necessary to remind their grandchild to eat and take insulin injections, these reminders could sometimes feel like restrictions or punishments. This feeling was particularly strong when their grandchild perceived these reminders as limiting their freedom or negatively impacting their daily routines. The grandparents felt a deep sense of responsibility and often struggled with finding a balance between providing essential support and respecting their grandchild’s autonomy. They were acutely aware of the emotional toll this balancing act took on their grandchild and themselves, and they navigated these challenges with a mix of determination, empathy, and guilt. When their grandchild didn’t finish a meal, they tried not to stress, instead offering alternatives later, with fruit often seen as a suitable solution.

“My son and his wife would like X to eat everything on his plate, they say he can’t always get what he wants. But, given his strong will and self-determination, I see no reason to force or stress. We sit for a while and talk, then we play a bit and after a while it’s usually okay to offer him a piece of fruit or a snack”. (Grandmother, 66 years).

The grandparents described having different roles in the family; some chose to engage fully in the self-management of their grandchild’s type 1 diabetes, while others stated that their tasks involved arranging practical matters such as transportation to school, cooking, or playing with their grandchildren.

“My X thinks I’m not getting involved enough in the disease itself, I truly want to learn more, but I’m so worried about making mistakes that will affect X. I’m much better at driving cars, so I usually drive and pick up our grandchild from training and school”. (Grandfather, 72 years).

The grandparents emphasized the importance of acknowledging their grandchild’s emotional needs and desire to feel “normal.” They spent time playing games, reading books, or simply talking about their grandchild’s interests and feelings, hoping to help their grandchild feel understood and valued. They involved their grandchild in decision-making processes related to self-management, allowing them to express their preferences and hopefully feel more in control.

“We try to let him be involved, so he presses the pump when I guide him, and he helps prepare his food. And when his blood sugar is high, he chooses which route we take on our walk. You can really see how much it means to him.” (Grandmother, 65 years).

#### Using but not fully trusting self-management technologies

The grandparents used the technologies but did not fully trust them. Most grandparents expressed more uncertainty about diabetes technologies than parents, largely because they used them less frequently and had fewer opportunities for hands-on learning. Even if modern technology allows a so-called tracking function to be connected to insulin pumps i.e. a phone application that enable grandparents to track their grandchildren’s blood glucose on their phones, they only used it when their grandchild was with them, except for a few people who to calm themselves couldn’t help but open it and check that everything was fine with their grandchild.

“The technology is amazing, but I still double-check everything. I guess trust comes with routine, and we don’t have that daily practice.” (Grandmother, 68 years).

Many grandparents talked about and wished for sharing the responsibility of their grandchild’s technical devices with a partner, expressing concern about what would happen if they could no longer manage it. They wished more family members could manage the technical parts of type 1 diabetes. Further, they couldn’t understand how other families managed to learn type 1 diabetes technologies so fast and without support. A grandparent described being the one who always cared for the technical parts during their grandchild’s visits, while the other grandparent looked after the siblings, showing reluctance to learn about the technical devices of type 1 diabetes. In some cases, grandparents expressed a desire to help out with the technical issues, but the fear of making a mistake and hurting or even killing their grandchild held them back.

When he’s with us, I look after the siblings. I want to learn more, but I’m honestly scared of doing something wrong. With diabetes I feel like one mistake could have serious consequences.” (Grandfather, 66 years).

The grandparents described being accustomed to having their grandchildren around, often for sleepovers. They didn’t want the disease or their concerns about the technical issues to put an end to it, so they decided that it had to be possible to make it work. However, they were worried about the insulin pump beeping at night. They feared that they might not hear the beeping, that they might not wake up, or that their grandchild could have such low blood sugar that they wouldn’t be able to wake them up. Nevertheless, they believed these situations would become easier over time. Additionally, the grandparents found it challenging to sleep well when the child stayed with them, and setting alarms for the child’s sleepovers was considered a necessary precaution.

“When he sleeps here with us, I stay in the room upstairs, acting as his device because I don’t fully trust them. If the pump beeps, I go in with a headlamp. Then I just give him insulin if it’s too high, and if it’s too low, I start with a sweet drink they prefer, because it’s better than dextrose. I don’t sleep well on those nights, but it’s nothing compared to the parents’ lack of sleep. (Grandmother, 69 years).

### Balancing expectations and own needs

While balancing expectations and their own needs, the grandparents struggled and had to handle a growing pile of concerns. Simultaneously, they were trying to hold the family together. It meant that while they were helping others, they also strived to maintain their activities in daily life to achieve such a balance.

#### Handling a growing pile of concerns

The grandparents felt anxious when their child was worried, and at times, they found it difficult to support both their child and grandchild at the same time. They were continually concerned about their child’s and grandchild’s mental and physical health, especially regarding potential complications from type 1 diabetes in their grandchild. They wanted to offer comfort and reassurance to their child, but they also needed to remain available for their grandchild, and they described honest communication as crucial but challenging.

“When someone is completely exhausted and stressed, it can be difficult to come up with a friendly suggestion without it being received as pure criticism, and I definitely don’t want to argue or start a fight.” (Grandmother, 59 years).

Several grandparents expressed that they often thought about and occasionally worried about the financial situations of their children and grandchildren, frequently due to sick leave caused by burnout. They were aware of the economic strain that managing Type 1 diabetes could place on a family. For instance, a grandparent shared concerns about their child’s frequent absences from work due to the need to care for their grandchild, which reduced the family’s income and financial concerns.

Regarding sick leave, a grandparent mentioned that the surroundings do not realize the impact that type 1 diabetes takes on parents, making it challenging for them to seek support from others, including uncomprehending bosses. They described how their child struggled to explain the demands of managing type 1 diabetes to their employer, who in some cases lacked empathy and understanding. This lack of support made it difficult for parents to take necessary sick leave without fear of others’ negative comments and additional financial loss.

“People around them don’t realize how demanding this disease is, and the employer certainly doesn’t. It’s heartbreaking to see them struggle without the support they need. (Grandmother, 70 years)”.

Issues of responsibility were another concern at school. Grandparents found their child having to advocate fiercely for their grandchild’s needs. A grandparent shared how their child had to attend several meetings with school staff to enable their grandchild to carry snacks or have access to a private space for changing insulin injection sets. For example, a grandparent told their child had to repeatedly educate school staff about type 1 diabetes management, including the importance of insulin administration and monitoring blood glucose levels. Frequent changes of resource personnel also posed significant challenges. A grandparent shared how their grandchild had to continually adapt to new aides or teachers, each with different levels of understanding and skill in managing type 1 diabetes. This inconsistency frequently disrupted the grandchild’s routine and increased stress for the child, the parents, and the grandparents.

“They have to fight for everything, with the school, with people who don’t get what type 1 diabetes really means. Meetings, explanations, new staff who don’t know what to do … it wears them down. I wish others could see how much responsibility they carry every day.” (Grandfather, 72 years).

#### Trying to hold the family together

The grandparents expressed a strong sense of responsibility to support and hold the family together. They did not find it difficult or hard for themselves and try to assist the grandchild with type 1 diabetes, his or her parents, and siblings as much as possible. For example, one grandparent regularly cooked meals and delivered them, ensuring that healthy food were served without adding extra stress to the younger generations already busy schedule. Some grandparents felt a strong need to stay in touch and keep track of what was happening with their children and grandchildren in case they needed to step in and help. In contrast, others indicated they completely trusted they would be contacted if assistance was necessary. The grandparents focus on being strong and positive for their family. However, there is always a lingering concern for their children and grandchildren in the background.

“Yes, yes, I’m still worried. It’s not easy to be a parent with a small child with type 1 diabetes and have to talk and discuss and try to get the resources they need, both in preschool and at school and so on. And so she, like so many others, was on sick leave for a period. So it’s clear that you’ve been kind of worried about both her and X”. (Grandmother, 67 years).

The grandparents emphasize the necessity of family communication in managing their grandchild’s type 1 diabetes. A parent noted that they were a close-knit family where open dialogue was the norm. Despite this understanding, there were occasional remarks and subtle comments that implicitly pointed to perceived unfairness. These comments were often made in passing but carried an underlying message of perceived inequality. These types of comments were described as the most difficult to address in discussions. A grandparent suggested that there might be some disappointment among the siblings, but the situation is already so challenging that no one has the energy to express themselves openly, and there is a certain shame associated with feeling jealous.

However, the grandparents found that the family was growing closer as they tried to support the child and each other. A grandparent reflected that parents may need to devote a significant amount of time and energy to managing their child’s type 1 diabetes, which can lead to less attention for siblings. Thus, the grandparents can serve as practical and emotional support for the siblings.

“This is what you have to deal with when someone in the family receives a diagnosis. You have to support each other. I am the kind of person who has always been strong, or what should I say. I have been told that for 20 years since my parents died very early, so I have been given the perception that I am strong all the time, and I manage it in a way…” (Grandmother, 64 years).

#### Maintaining personal activities

Most grandparents described an active retirement lifestyle. A few grandparents explained that their activities were adjusted to meet the needs of the family, reflecting their role as flexible and supportive rather than primary caregivers. They enjoyed having lunch with friends, dining with fellow retirees, and going to the movies. A grandparent discussed the joy of volunteering with friends, and another the pleasure of physical activities alone or in company with other retirees. The grandparents found great joy in social interactions and appreciated their retirement lifestyle. Despite their busy schedules, they always found time to support their grandchildren with type 1 diabetes and their family. They were keenly aware of their grandchild’s parents’ challenges and strived to help without imposing demands. They wanted to be there for their grandchild and family, providing support and comfort. Despite their own commitments, the grandparents offered a listening ear, a comforting hug, and a reassuring presence, doing everything they could to help their grandchild and family navigate their way forward.

“We try to live our lives as usual, but of course we adjust when the family needs us. It’s natural — that’s what grandparents do.” (Grandmother, 64).

The family situation affected the activities and leisure time of some grandparents’ social interactions, as the family’s schedule shifted to revolve around the type 1 diabetes child’s needs. Nevertheless, the grandparents repeatedly expressed that having plenty of time allows them to help their grandchild with type 1 diabetes and family, and they find joy in doing so. They expressed cherishing the time spent together and the opportunity to be actively involved in their grandchild’s life. They enjoyed going on trips and participating in activities with their grandchild, who had type 1 diabetes. A grandparent gave examples of ski holidays, weekend fishing, and cottage visits. They claimed they had the financial means to assist if needed, despite the parents reassuring them that they did not have to feel obligated to do so. A grandparents felt fortunate to be able to dedicate their time and energy to supporting their family.

“I’m retired, healthy, and have a lot of leisure time, so I can spend time with both my friends and my grandchildren. I also have a different financial situation compared to them; I want to help, and I can. It just feels so good.” (Grandmother, 69 years).

## Discussion

The main theme of the results described perceptions of being grandparents of children living with type 1 diabetes as being the lighthouse that lights up dark paths, but still in need of attention to keep shining. Additionally, the grandparents can also be illustrated as lighthouse keepers. The grandparents grasped overwhelming situations, took on new responsibilities, and pushed the margins. They used but did not fully trust the technologies, illustrated as lighthouse keepers who make sure that the complex systems operate safely. The grandparents handled a growing pile of concerns and tried to hold the family together, related to lighthouses having large responsibilities to navigate. As well as lighthouse keepers, the grandparents needed to maintain personal activities to sustain function and health. Viewing the findings through an ecological systems perspective (21) clarifies how grandparents’ experiences are embedded within broader family dynamics. Their emotional responses, responsibilities, and boundaries are influenced not only by their relationship with the grandchild but also by their interdependence with the child’s parents, school environments, and healthcare actors. Time emerged as an important dimension across the themes. As grandparents gained more experience, their confidence grew, their worries changed, and their ways of supporting the family developed. This ongoing process fits with ecological views where adaptation and competence grow through repeated caregiving interactions system. This temporal adaptation aligns with previous research showing that families of children with type 1 diabetes develop competence and stability through ongoing interactions and accumulated caregiving experience (24, 25).

Related to managing the unknown, grandparents were deeply moved by grandchildren living with a lifelong disease and its demands. Novak-Pavlic (2022) noted that a grandchild’s unexpected illness profoundly affects grandparents’ emotional health. They emphasized involvement in self-management and providing practical and emotional support. The discussion highlighted their roles in diabetes management and the importance of acknowledging the child behind the disease, aligning with person-centered healthcare (Ekman et al., 2011). Recognizing people with lifelong diseases as humans with emotions and desires is crucial in both general and medical care.

As mentioned earlier, grandparents had to adapt to new responsibilities and push their own limits, both practically and emotionally. They learned to operate complex self-management technologies, such as insulin pumps and continuous glucose monitors, even if they initially mistrusted these devices and, more importantly, their own ability to use them. The fear of making mistakes that could harm their grandchild was a significant concern. However, over time, a sense of confidence grew as they became more familiar with the technology and their theoretical knowledge increased. The grandparents’ adjustment to the grandchild’s condition was characterized as gradual and emotionally challenging. Adjusting to a grandchild’s illness has previously been compared to that of parents, and the similarities between them highlight how severely the grandchild’s illness impacts the grandparents (4, 26). Nonetheless, consistent with (3), our findings indicate that the challenges faced by grandparents differ from those experienced by parents. The uniqueness of the grandparents’ situation stems from their experience of double grief, related to both their child and grandchild (27, 28). Another important aspect of the grandparental role is caring for the entire family, providing support to various family members in different ways (28, 29). The grandparents in this study felt they needed to accept a new reality, and managing the related situations became easier over time, suggesting a potential process of reconciliation. In conclusion, given the significant practical and emotional impact on grandparents and their supportive roles, they should be included in the family-centered care provided to children living with type 1 diabetes. The International Society for Pediatric and Adolescent Diabetes [ISPAD] (30) emphasized the importance of familial and social support systems for children with type 1 diabetes but did not specifically identify grandparents as having a significant role in the clinical practice consensus guidelines for psychological care.

Our study showed grandparents see their support as meaningful despite challenges. While caring for children with long-term diseases has negatives, it also brings positives, such as stronger family bonds, gratitude for healthcare, and admiration for the child’s resilience. These grandparents felt empowered, focusing on living fully, and appreciated being involved in their grandchildren’s lives. Concerning the experiences of trying to hold the family together, the grandparents expressed how they provided major support to their families. In another study (31) related to pediatric cancer, grandparents noted that their children had unrealistic expectations of them. The grandparents in our study did not express feelings of not being viewed as supportive enough; however, they conveyed challenges in balancing expectations with their own needs. The participants in this study raised concerns about the children feeling exhausted due to the demands of type 1 diabetes self-management and the associated anxiety, as illustrated when friendly suggestions were perceived as criticism. Compared with other long-term illnesses, grandparents of children living with pediatric cancer voiced significant concerns about their daughters and sons not taking care of themselves and the long-term impacts the disease will have on their health (27, 32).

However, there are situations where significant involvement of grandparents might not be as welcome. According to Bol and Kalmijn (33), some families may encounter tension when grandparents assume roles that parents believe should be theirs. This can lead to conflicts, especially if grandparents start to overstep boundaries and take on the parental role. Such dynamics can create stress and disrupt the balance within the family, potentially resulting in disagreements about the best way to manage the child’s disease (33). Conversely, grandparents may also feel uncomfortable being forced into the parental role (32). The grandparents’ perception of being a yes-person was challenged when they had to care for a sibling for an extended period while parents focused on the child living with a long-term disease, such as during the hospitalization of children with pediatric cancer (32).

In summary, these results showed that the participants grappled with the existential aspects of being grandparents to children living with type 1 diabetes. The grandparents worried that even small mistakes in self-management could lead to serious health consequences for the child, such as injuries or even death. They also expressed fear of finding their grandchildren dead due to hypoglycemia. In a study aimed at exploring the experiences of being grandparents to children living with pediatric cancer, a grandparent illustrated the natural process of grandparents dying before their grandchildren; however, this severe disease, which has serious health consequences, calls into question this natural generational process and creates a significant existential impact (32).

The grandparents in this study shared their perceptions of young children who are forced to depend on medical devices throughout their lives, a view that can be linked to existential insights when comparing the grandparents’ seniority and extensive life experience to a young child who has her whole life ahead of her. In Holmer et al. (31), a grandmother describes how her advanced age has contributed to her resilience, viewing it as a strength. The process of developing resilience can occur naturally with aging, as individuals accumulate experiences and coping strategies over the years (31). This resilience, developed over a lifetime, may help grandparents cope with the challenges of having a grandchild living with type 1 diabetes.

### Limitations

A limitation of this study was that most grandparents lived near their grandchildren, which is not the case for many grandparents around the world. Including more grandparents who live far from their grandchildren, such as those residing in different parts of the country or in other countries, might yield different results. Another limitation of this study was the lack of cultural diversity among the participants, as none of the grandparents were born outside of Sweden. Additionally, most participants were women, which may have influenced the results. While men and women can have different experiences, this study did not focus on that aspect.

### Clinical implications

Our results highlight the importance of providing education and support to grandparents. Both pediatric and adult care emphasize the value of an extended social network when caring for young individuals living with type 1 diabetes. Therefore, we should include grandparents who are eager to feel secure in supporting their grandchild. To empower grandparents as practical and emotional supporters in self-management, they should be integrated into self-management education within healthcare, with the goals of promoting the well-being of young individuals living with the disease and easing the responsibilities, stress, and burden on parents.

Recognizing the emotional and practical challenges faced by grandparents of children with type 1 diabetes remains largely unexamined in research. In the literature search for this publication, we found a limited number of studies focusing on this group, highlighting the need for research on these previously overlooked family members. Addressing this research gap will directly impact the lives of affected families and all their members. Previous studies suggest that offering structured education programs may positively influence grandparents’ stress and worry and also contribute to the level of functional and emotional support that grandparents can provide families (34, 35).

## Conclusion

The role of grandparents in families with children living with type 1 diabetes is multifaceted. They take on significant responsibilities, including learning complex self-management technologies and providing emotional and practical support. Their involvement is crucial for maintaining the family’s stability and ensuring the child’s well-being. However, this role comes with challenges. Grandparents face existential concerns, fearing the severe consequences of potential mistakes in diabetes management. They experience double grief, mourning both their child’s and grandchild’s struggles with the disease. Despite these challenges, many grandparents exhibit remarkable resilience and are eager to assist their families in both large and small ways. Grandparents play an active and highly involved role in the family system and are affected by their grandchild’s disease in various ways. Despite being significantly impacted, grandparents mainly rely on informal support and information from non-professionals, such as their child, partner, or patient organizations.

## Data Availability

The raw data supporting the conclusions of this article will be made available by the authors, without undue reservation.
